# Retinal Detachment in the eye of Albino - A review

**DOI:** 10.22336/rjo.2026.05

**Published:** 2026

**Authors:** Abdullah Al Marshood

**Affiliations:** 1 Department of Ophthalmology, College of Medicine, Qassim University, Saudi Arabia

**Keywords:** retinal detachment, oculocutaneous albinism, macular hypoplasia, ocular hypopigmentation, OCA = Ocular manifestations in oculocutaneous albinism, RD = retinal detachment, VEPs = visually evoked potentials, RRD = rhegmatogenous retinal detachment, ARD = albino retinal detachment, PVR = proliferative vitreoretinopathy, OCT = Optical coherence tomography, BCVA = best-corrected visual acuity, PPV = pars plana vitrectomy, RPE = retinal pigment epithelium

## Abstract

Ocular manifestations in oculocutaneous albinism (OCA), a hereditary condition, are well documented. Retinal detachment in individuals with OCA is uncommon and poses challenges for retina specialists in terms of diagnosis and management. We reviewed the literature on the presentation and management of retinal detachment (RD) in oculocutaneous albinism (OCA). Identifying retinal breaks in the absence of retinal and choroidal pigments can be challenging. Chorioretinal sealing around retinal holes and breaks achieved through photocoagulation and/or diathermy is less effective compared to that achieved by cryopexy. Intraocular tamponade may also need to remain in the eye for an extended duration, and its side effects may affect the functions of the viable retina. The presence of macular hypoplasia and significant visual function impairment in OCA complicates the evaluation of the functional success of retinal detachment surgeries. More research, both in vivo and in vitro, is needed to identify effective management strategies, including low-vision rehabilitation and monitoring protocols, and to assess the impact of interventions for retinal detachment in OCA.

## Introduction

### Albinism and its magnitude

Albinism refers to a group of hereditary conditions characterized by a lack of melanin pigments in tissues such as the skin, hair, and eyes, which originate from the ectoderm [1]. When both the skin and eyes are affected—a common presentation—it is called oculocutaneous albinism (OCA). The prevalence of OCA ranges from 1 in 17,000 to 1 in 20,000 individuals [2]. There is significant variation in the prevalence of OCA across subtypes.

### Types and Underlying Causes of albinism

Albinism can occur in isolation or as part of a syndrome that affects other systems. Oculocutaneous albinism (OCA) is genetically determined and typically presents within the first year of life. There are seven types of OCA based on the mutation site. The most common type is OCA2, which is highly prevalent among Sub-Saharan Africans (1:3,900). OCA2 is less common among all Americans (1:36000) compared to African Americans (1:10,000). OCA5, OCA6, and OCA7 are very rare. OCA1 is most prevalent in America and China, with a rate of 1:40000, and it accounts for 70% of all OCA cases [2-4]. Isolated ocular albinism is an X-linked condition that occurs in 1:60,000 males [5]. The prevalence of isolated ocular albinism is 1:50,000 [2,6]. Albinism can also be associated with Hermansky-Pudlak syndrome (HPS), Chediak-Higashi syndrome, Angelman syndrome, and Prader-Willi syndrome [7,8]. These syndromes are more prevalent than OCAs.

Melanocytes develop embryologically from the neural crest and are found in the skin, eyes, hair, and inner ear. They make up 5% to 10% of the basal cells in the epidermis. Melanin is also produced by retinal pigment epithelial cells. Two types of melanin pigments, eumelanin and pheomelanin, are generated in melanocytes with the help of melanosomes. Eumelanin protects cells from ultraviolet radiation type B (UVB), while pheomelanin provides no UV protection. In OCA, melanin production is impaired. The main reason for the decreased melanin is a shortage of the enzyme tyrosinase. In syndromic albinism, melanin synthesis is not always affected. Hypomelanosis is the underlying cause of OCA symptoms, mainly affecting the skin, hair, and uveal tissues in humans [2,3,9,10].

### Ocular manifestations

In albinism, the eyes and visual functions are primarily affected [11]. Hypoplasia of the fovea, which leads to reduced visual acuity, occurs due to abnormal retinal cell development, thinning of the outer nuclear layer, tightly packed Henle nerve fiber layer, and increased foveal cone density [12]. Nystagmus, either of the pendular or jerky type, develops during the first few weeks of life in individuals with Oculocutaneous Albinism (OCA). Although it diminishes with age, strabismus is commonly observed. A spontaneous compensatory head posture and a null-point gaze help individuals with OCA mitigate the impact of nystagmus on their visual function. However, this condition negatively affects their reading skills [11,13]. People with OCA are predominantly hyperopic or may experience increasing astigmatism as they age. An ocular examination reveals hypopigmentation of the iris and retinal pigment epithelium, resulting in blue or light brown irises and defects in iris transillumination. Fundus evaluation shows enhanced visibility of deep choroidal vessels. The degree of pigmentation defects varies by albinism subtype. Additionally, individuals with OCA often experience photophobia. In the intracranial pathway, particularly at the optic chiasm, misrouting of optic nerve fibers in those with OCA, as measured by monocular visually evoked potentials (VEPs), shows bilateral contralateral predominance [14]. Bridge et al. studied the morphology of the human brain in individuals with OCA. They found a difference in cortical thickness within V1, a region of the right hemisphere’s posterior ventral cortex of the occipital lobe, compared to individuals without albinism. They also noted a negative correlation between visual acuity and cortical thickness in V1, suggesting that reduced vision may be associated with decreased cortical thickness in individuals with albinism [15].

### Vision-related quality of life and visual function derangement

Impaired distance vision, its impact on mental health, and the challenges faced in daily living are the primary factors negatively affecting the vision-related quality of life for individuals with OCA [16]. Low vision combined with aesthetic taboos from skin changes leads to social stigma for oculocutaneous albinos [17]. More than three-fourths of albinos experience a severe grade of photosensitivity, which is related to fundus hypopigmentation but not iris involvement in OCA [18]. This restriction limits their outdoor work and mobility; however, many have noted significant improvements in their visual function when using filters.

Most non-syndromic albinos have a life expectancy similar to that of the non-albino population. However, albinos in Africa are at a higher risk of skin cancer and live an average of 40 years [19]. Consequently, middle- and old-age-related systemic health issues, such as diabetes, degenerative diseases, and inflammatory diseases, along with their ocular manifestations, could also affect albinos. The heterogenized vitreous humor in old age puts additional stress on the retina, potentially leading to retinal detachment or macular holes [20]. The eyes of albinos undergoing cataract surgeries or experiencing advanced diabetic retinopathy are particularly vulnerable to RD. Phacoemulsification cataract surgery increases the risk of rhegmatogenous retinal detachment (RRD) by 1.7. [21]. Nystagmus, a common feature in OCA, heightens the risk of vitreous disturbances, which may also contribute to retinal detachment [22]. Retinal detachment in OCA, known as albino retinal detachment (ARD), poses challenges for diagnosis and effective management. Therefore, it is essential to explore different methods of ARD assessment and management outcomes.

### Objective of the review

We reviewed the current literature on retinal detachment and its management in persons with OCA.

## Methods

### Literature search

We used “OCA” + “retinal detachment” + “Human” words to search for articles. We used search engines such as PubMed, Google Scholar, Scopus, the Cochrane Library, ScienceDirect, and OpenMD to identify relevant published articles. We applied a language filter for “English” to retrieve full articles or abstracts on these topics. Additionally, we limited the article search to those published in the last 50 years and examined in detail all articles on the topic published since 2000. Duplicates from different search sites were removed. The algorithm for collecting research articles is presented in **Fig. 1**. We employed the GRADE criteria to evaluate the quality of evidence in the published literature on the topic [23]. Furthermore, we used the Critical Appraisal Toolkit (CAT) to assess the evidence for the review topic [24].

## Results and discussion

To the best of our knowledge, the earliest case of ARD reported in the literature was in 1999 [25]. Both diagnostic and surgical techniques have improved since then. However, publications detailing ARD are limited, primarily limited to RRD in OCA [22,25-29]. They consist of case series or case reports, resulting in low-quality evidence. The focus is mainly on surgical outcomes and challenges. In this review, the challenges and interpretation of ARD-related information, along with conventional management of RD, were presented to guide retina specialists in managing such cases.

### Rhegmatogenous retinal detachment in OCA Presentation

Typically, cases of RRD present with new photopsia and vitreous floaters. An alert patient might report a constricted visual field in the affected eye. There is a gradual, progressive loss of vision, especially when the macula is detached [30]. Someone with OCA already experiences reduced visual acuity, color vision impairment, and reduced contrast sensitivity [11]. Therefore, the initial ophthalmic workup for ARD may differ somewhat from standard RRD evaluations. Distance visual acuity should be assessed using non-illuminated charts, or by having the patient wear filter glasses they usually use indoors. Comparing these results to previous measurements and the fellow eye can help gauge how ARD affects vision. Although field testing can be difficult with nystagmus, it provides caregivers with insights into ARD’s effects and the impact of foveal hypoplasia and visual pathway misrouting [11,31]. A thorough history for ARD should include the duration of decreased visual acuity, any previous ocular trauma, prior eye surgeries, and episodes of eye hemorrhage. A comprehensive systematic review will aid in the diagnosis of secondary ARD and syndromic ARD.

### Diagnosis

The goal of the physical and ocular examinations in cases of ARD is to detect all retinal breaks, select the most suitable method of chorioretinal adhesion, and monitor the impact of the intervention more frequently and for a longer duration. It is essential to note all ocular and systemic comorbidities that may compromise visual acuity, enabling a more accurate evaluation of the results of ARD surgery.

For the past 40 years, Lincoff’s rules for assessing rhegmatogenous retinal detachment (RRD) cases have been relevant and judiciously followed in adults with RRD, thereby enhancing the success of retinal detachment surgery [32]. A greater number of breaks, inferonasal retinal breaks, retinal detachment extending into three or four quadrants, and the presence of proliferative vitreoretinopathy (PVR) and macular retinal detachment are known risk factors for surgical failure in the treatment of uncomplicated RRD [33]. In ARD, these tried-and-true methods for detecting retinal breaks often present challenges. This is primarily due to poor visualization of retinal breaks resulting from a lack of contrast between the retina and the choroid [22,26]. The absence of retinal pigment in the retinal epithelium fails to provide adequate contrast between the retinal layer and the retinal pigment epithelium around breaks, leading to missed detection of retinal breaks [11,34]. Additionally, the presence of nystagmus in OCA makes retinal assessment using a slit lamp bio-microscope challenging. Indirect binocular ophthalmoscopy is preferable to slit lamp examinations; however, using a wide-field contact lens has been shown to reduce nystagmus amplitude, allowing for proper retinal evaluation in OCA [22].

### Investigations

Although clinical assessment is sufficient for preoperative identification of retinal breaks, performing ultrasound scans of eyes with ARD is beneficial. These scans assist retina surgeons in detecting unusual astigmatic posterior segments and lesions behind detached retinas, such as retinoblastoma or melanosis. Optical coherence tomography (OCT) is not routinely required to evaluate macular status, as this can be determined through best-corrected visual acuity (BCVA) and clinical examination. Preoperative BCVA predicts potential postoperative BCVA [35]. In eyes affected by OCA, there is often foveal hypoplasia, leading to reduced distance vision. Therefore, the presence of fluid in the macula cannot be determined solely by BCVA and clinical examination. Consequently, OCT is recommended in these exceptional cases to evaluate the macula preoperatively and to predict functional outcomes. OCT can also detect PVD, which influences the surgical approach. During retinal surgery, the use of trypan blue has facilitated the identification of all retinal breaks in ARD [36].

### Surgical management of ARD

In conventional RRD, pars plana vitrectomy (PPV), laser application around all retinal breaks, and the insertion of vitreous substitutes such as silicone oil and inert gases are routinely performed steps in retinal detachment surgery to keep the retina opposed to the choroid [37,38].

To relieve vitreoretinal traction and support retinal tears, scleral buckling is used. This is complemented by cryopexy around all identified retinal breaks to achieve adequate chorioretinal adhesion. The latter is an older method, and if performed by an experienced surgeon, it provides very effective outcomes [39]. A single break in the upper area of the retinal pneumatic retinopexy is recommended.

In eyes with ARD, conventional methods of chorioretinal adhesion around retinal breaks, such as laser treatment, do not provide adequate sealing. Mansour et al. reported a series of 17 eyes with ARD. In three of five eyes in which endo-laser was used, RD recurred because adhesions around the retinal breaks could not be achieved [22]. In their case report, Henson et al. showed failed attachment when a retinal break was sealed with an endolaser [40]. However, Hiroshi et al. utilized a Krypton laser and achieved the desired adhesion and success [25]. Several reasons are postulated for the residual subretinal fluid after chorioretinal adhesion in ARD. Insufficient L-DOPA levels in RPE cells of albino individuals and faulty aquaporin-1 channels are among the explanations provided by researchers [41,42]. Cryotherapy to seal retinal breaks and apply a permanent scleral buckle has shown promising results in treating ARD [22,28,43]. Although OCA eyes are typically small due to developmental defects from the absence of melanin in the RPE and choroid, the vitreous needs attention, as PVR is common. Posterior vitreous detachment and PVR require management, and silicone oil serves as a substitute for vitreous following pars plana vitrectomy in both eyes with RRD in OCA and those with secondary RD after PDR, tumors, and trauma. The heavy silicone oil should be used and retained for an extended period, as nystagmus could disrupt vitreoretinal adhesion and the apposition of retinal layers to the choroid [22]. The tamponade created by heavy silicone oil significantly reduces intraocular currents, thus preventing fluid entry through the break until the chorioretinal adhesion occurs [44].

### Postoperative care

The convalescence period is longer after RD surgery for ARD compared to the conventional RRD surgery for non-albino individuals. This is because eye movements caused by nystagmus interfere with resting retinal tissues and hinder the absorption of subretinal fluid. Manual tapping of subretinal fluid through the sclera with diathermy during the scleral buckling procedure may be beneficial when RPE fluid pump activity is absent in ARD [45]. Postoperative patient positioning and immobilization of the eye using fixation sutures are methods that have been attempted to reduce fluid accumulation and the recurrence of RD [46,47].

Among 17 eyes with ARD in a series, 9 eyes (53%) experienced retinal detachment recurrence after the initial surgery, and in 3 additional eyes, RD resolved after the second surgery. Four eyes required 3 to 5 surgeries. The average BCVA improvement was 4.5 lines, observed 77 months after the initial surgery. Thus, it appears that anatomical success in managing ARD was delayed in some cases, requiring multiple interventions; however, visual recovery was significant. Interestingly, 2 cases with intravitreal hemorrhage upon further examination were found to have syndromic OCA [22]. Therefore, a comprehensive investigation and genetic profiling of OCV are recommended before managing ARD. In another series of ten patients with ARD, Sinha et al. observed that four eyes treated with cryopexy and scleral buckling showed no recurrence at 12 months. In contrast, in 6 eyes treated with PPV, two experienced recurrence after 5.5 months. In the SB group, vision improved from a mean of 2.15 logMAR to 1.24 logMAR, while in the PPV group, it improved from 2.63 logMAR to 1.35 logMAR. The authors also noted that fellow eyes of individuals with OCA had low vision disability, but none developed RD during follow-up [26].

### Management outcome

Mansour et al. noted that in their series of 17 patients with ARD, 9 eyes had RD with the macula on, and 8 had RD with the macula off. Those with the macula on at the time of surgery experienced severe visual impairment, whereas those with the macula off saw their vision improve to 20/200 or better in most cases [22]. These findings conflict with the conventional understanding of a negative correlation between macular on- and off periods and visual recovery after RD surgery. Further research with a larger sample size is recommended to explore the relationship between vision and macular involvement in RD. Graven et al. noted that in eyes with RD and the macula off, visual recovery is good if managed within the first 3 days of macular detachment [48]. Perhaps this cohort of 17 eyes with ARD was operated on promptly, and therefore, the correlation between macular involvement and visual recovery was unpredictable [22].

### Secondary ARD due to diabetic retinopathy

The prevalence of diabetes and diabetic retinopathy is high. DR is expected to remain elevated, especially among the diabetic populations of the Middle East, North Africa, and the Western Pacific regions [49]. Those with OCA in these areas are also at a higher risk for ARD secondary to proliferative diabetic retinopathy. PDR-related RD in individuals with OCA has been documented in a few cases 40,43,50,51. Due to the lack of pigments in the RPE, Panretinal photocoagulation (PRP) is challenging and often fails to achieve the desired effect. Surgical management of ARD also requires expertise in carefully removing proliferative vitreoretinal adhesions, using heavy silicone oil or gas for tamponade, and controlling systemic risk factors. The visual improvement and anatomical success in these cases tend to be short-term. Since it is a bilateral condition, it presents a challenge for both the surgeon and the patient.

### ARD secondary to ocular tumors

The literature reports few cases of retinoblastoma and melanoma leading to retinal detachment in individuals with OCA [52,53-55]. OCT and sonography of the posterior segment are essential for diagnosing such secondary ARD. The lack of melanin pigment in OCA patients complicates the clinical diagnosis of this condition. In these cases, the anatomical and functional outcomes are often poor, and preserving the eyeball may not be feasible.

### RD in syndromic OCA

The OCA-related syndrome is well documented, and genetic studies of such cases have been reported in the literature. Patients with Chediak-Higashi Syndrome have oculocutaneous albinism along with immunodeficiency and a mild bleeding tendency [56]. Hermansky-Pudlak Syndrome presents as OCA with a bleeding diathesis, and, in some individuals, includes pulmonary fibrosis, granulomatous colitis, and/or immunodeficiency [57]. These patients have a shortened life expectancy, and ARD is not common among them. There are syndromes associated with retinal detachments, such as Knobloch syndrome, that, when observed in a person with OCA, can be associated with ARD [58,59]. Often, such cases with ARD present with vitreous hemorrhage or retinal bleeding during or after RD surgery. Therefore, obtaining a genetic history, as well as information about bleeding and bruising tendencies, should be asked about before starting management of ARD. Managing ARD in syndromic OCA is similar to managing RRD. Extra care should be taken to control bleeding.

### Low vision rehabilitation of OCA post ARD

Low-vision rehabilitation strategies are essential for improving vision-related quality of life for individuals with OCA. As many as 6.1% of children under 18 who sought multidisciplinary services in the Netherlands within a year had OCA [60]. Binocular vision, accommodation difficulties, and poor contrast sensitivity were the main orthoptic issues observed in children with OCA [61]. After managing ARD, visual function should be reassessed in individuals with OCA, and rehabilitation services should be adjusted accordingly [62].

### Limitations

The current review had several limitations. Due to the low level of evidence, the literature, including case series and case reports on ARD, could not provide conclusive support for changes in management practices. Further research with larger sample sizes and longitudinal or interventional studies on ARD management is required.

## Conclusions

Diagnosis and management of retinal detachment in persons with OCA pose challenges for retina surgeons. Locating retinal breaks is difficult due to the lack of pigmentation in the RPE and choroid. Poor adhesion around the retinal break from laser treatment, slow or absent resorption of subretinal fluid, and nystagmus preventing effective tamponade result in anatomical and functional failure of RD surgery in OCA. The effectiveness of intervention for ARD is also hard to assess, as visual function is already impaired by foveal hypoplasia and the higher visual center effects of OCA. Lifelong follow-up and regular review of low-vision rehabilitation strategies are necessary to manage ARD patients.
